# Actin Cytoskeleton Manipulation by Effector Proteins Secreted by Diarrheagenic *Escherichia coli* Pathotypes

**DOI:** 10.1155/2013/374395

**Published:** 2012-12-30

**Authors:** Fernando Navarro-Garcia, Antonio Serapio-Palacios, Paul Ugalde-Silva, Gabriela Tapia-Pastrana, Lucia Chavez-Dueñas

**Affiliations:** Department of Cell Biology, Centro de Investigación y de Estudios Avanzados del IPN (CINVESTAV-IPN), Apartado Postal 14-740, 07000 México, DF, Mexico

## Abstract

The actin cytoskeleton is a dynamic structure necessary for cell and tissue organization, including the maintenance of epithelial barriers. Disruption of the epithelial barrier coincides with alterations of the actin cytoskeleton in several disease states. These disruptions primarily affect the paracellular space, which is normally regulated by tight junctions. Thereby, the actin cytoskeleton is a common and recurring target of bacterial virulence factors. In order to manipulate the actin cytoskeleton, bacteria secrete and inject toxins and effectors to hijack the host cell machinery, which interferes with host-cell pathways and with a number of actin binding proteins. An interesting model to study actin manipulation by bacterial effectors is *Escherichia coli* since due to its genome plasticity it has acquired diverse genetic mobile elements, which allow having different *E. coli* varieties in one bacterial species. These *E. coli* pathotypes, including intracellular and extracellular bacteria, interact with epithelial cells, and their interactions depend on a specific combination of virulence factors. In this paper we focus on *E. coli* effectors that mimic host cell proteins to manipulate the actin cytoskeleton. The study of bacterial effector-cytoskeleton interaction will contribute not only to the comprehension of the molecular causes of infectious diseases but also to increase our knowledge of cell biology.

## 1. Introduction

Epithelial layers are essential to keep normal organ operation by creating boundaries to the movement of ions and molecules. This event allows the formation of different tissue compartments and ion gradients that drive transport across the epithelium. The tissue compartments include kidney tubules, ducts within the liver, and the lining of the gastrointestinal tract and lungs. In many of these tissues, the epithelium is also a barrier between the organ tissue and the external environment, representing the first layer of defense against pathogens. Epithelial cells form sheets by binding to each other through apically located adherent junctions (AJs) and more basally located desmosomes [1]. Polarized epithelial cells are characterized by the separation of distinct apical and basolateral domains that sort, traffic, and localize unique subsets of plasma membrane proteins. Epithelial cells undergo polarization through crucial interactions with the actin cytoskeleton and associated signaling molecules, resulting in the formation of junctional complexes. The apical plasma membrane surfaces of polarized epithelial cells are deeply associated with the actin cytoskeletal network [2]. In the case of absorptive intestinal epithelia, the apical cell surface (the intestinal lumen) is composed of finger-like projections called microvilli. Microvilli are comprised of parallel actin bundles that anchor to the subapical actin network through direct interactions with several actin bundling proteins. The ezrin, radixin, and myosin (ERM) family of actin-binding proteins provides stability to the microvilli and these proteins are important components of epithelial cell architecture as they provide a link between the cortical membrane and the actin cytoskeleton [3].

The most commonly described cellular target of pathogens is the cytoskeleton. Various intracellular microorganisms harness cytoskeletal components to gain entry to and to propel themselves within host cells [4]. The cytoskeleton of eukaryotic cells is composed of actin filaments, microtubules, and intermediate filaments. In terms of bacterial pathogenesis, the most extensively studied of these are actin filaments [5].

## 2. Actin Cytoskeleton

The shape and movement of cells, as well as phagocytosis, intercellular communication and the distribution of organelles depend on actin [6]. Actin persists in the cell as two different forms: monomeric globular actin (G-actin) and polymeric filamentous actin (F-actin). Actin is one of the most abundant proteins in eukaryotic cells and is composed of 375 amino acids forming a single chain of 42 kDa. Its atomic structure was first solved for its complex with deoxyribonuclease I [7]. G-actin is a flat molecule of about 50 × 50 × 35 Å. Under physiological salt conditions purified monomeric or G-actin polymerizes to its filamentous form, F-actin. G-actin contains firmly bound one molecule of ATP that is hydrolyzed to ADP and Pi after incorporation into a growing F-actin filament. The ADP remains attached to the actin subunit, whereas the Pi dissociates slowly from the filament generating two filament ends with actin subunits differing in their bound nucleotide: either ATP or ADP [8]. Throughout polymerization, ATP-bound G-actin preferentially associates to the end containing ATP-actin subunits, the fast growing end, which has also been termed the plus or barbed end. After reaching equilibrium, actin monomers associate to the barbed end and an identical number dissociates preferentially from the opposite end, which has also been termed the minus or pointed end. Thus, under these conditions and in the presence of ATP actin subunits constantly associate to the barbed end and travel through the whole filament until they dissociate from the pointed end [9]. This event has been termed treadmilling or actin cycling and represents the sole basis for force generation for a number of motile processes [10].

 In eukaryotic cells, monomeric G-actin is in dynamic equilibrium with polymerized F-actin. Polymerization is regulated by capping proteins (such as gelsolin) at the plus ends, through the binding to these ends with high affinity and prevents elongation. Rapid signal-induced actin polymerization, including that which occurs during host-pathogen interactions, can be triggered by different mechanisms, including *de novo* nucleation that can involve the Arp2/3 (actin-related protein 2/3) complex [11]. Actin nucleation by the Arp2/3 complex, which is essential for phagocytosis, cell polarity and migration, forms an orthogonal Y-branched network of F-actin [12]. The Arp2/3 complex itself consists of seven proteins; it binds ATP and is activated by nucleation-promoting factors from WASP (Wiskott-Aldrich syndrome protein) family including WASP, neural (N-)WASP and three SCAR/WAVE (WASP family verprolin homologous protein) isoforms, as well as other factors such as cortactin and the actin-binding protein Abp1p [13]. WASP family proteins possess a C-terminal Arp2/3-binding and activation domain (WCA domain). At the N terminus, WASP and N-WASP proteins contain a WASP homology 1 (WH1) domain, which is related to a domain typical of the ENA/VASP (Ena/vasodilator stimulated phosphoprotein-like protein) protein family (also known as the EVH1 domain), and a Cdc42-interactive GTPase-binding domain (GBD) motif, which preferentially binds Cdc42. In addition, these proteins possess a phosphatidylinositol bisphosphate (PtdIns(4,5)P2)-binding region (B) adjacent to the GBD domain. The WCA domain increases actin nucleation by Arp2/3 by about 80-fold. WASP family proteins are autoinhibited by binding between the central region of the WCA domain and the B/GBD region, which is also near to the CRIB (Cdc42/Rac interactive binding) domain. This autoinhibition can be released by binding of GTP-bound Cdc42 to the GBD domain [14]. Cdc42 can thereby directly activate actin nucleation [11].

Filament formation is promoted and stabilized through the action of proteins such as profilin and cortactin, and the filament is depolymerized through the action of proteins such as cofilin or gelsolin [15]. Actin filaments (also called microfilaments) also bundle with other actin-interacting proteins, including fascins [16], forming more substantial structures. Alternatively, the filaments can be cross-linked by branching, which is initiated by actin-nucleating proteins [17] to form a meshwork such as cortical actin. F-actin fibers form the microfilament network inside the cell, varying from myosin-containing contractile stress fibers to the cortical actin network that resides beneath the plasma membrane and around intracellular organelles. Actin fibers are also used to make sheet-like extensions, such as lamellipodia, membrane ruffles and blebs; finger-like protrusions, such as microvilli and filopodia; or dot-like podosomes. These structures are modified by the action of several actin-binding and signaling proteins [18]. The actin cytoskeleton is highly dynamic and is mainly manipulated by members of the Rho-family GTPases that control signal transduction pathways linking membrane receptors to the cytoskeleton. Rho-family GTPases regulate several cellular processes, including F-actin polymerization, assembly of intercellular junctions, cell polarity, cell migration, and membrane trafficking [19]. The Rho GTPases belong to the superfamily of Ras proteins. To date, more than 20 different mammalian Rho GTPases have been identified, which are ~50% homologous to each other and ~30% similar to Ras [20]. Rho proteins function as molecular switches in many eukaryotic signal transduction pathways and, most importantly, regulate the actin cytoskeleton. The switch function of Rho proteins depends on their regulation by a GTPase cycle. Rho GTPases are inactive when bound to GDP and are associated with a guanine nucleotide dissociation inhibitor (GDI). They are activated by the GDP/GTP exchange caused by guanine nucleotide-exchange factors (GEFs; ~60 have been recognized to date), which themselves are controlled by extracellular-induced signaling. GTP-bound Rho proteins induce many downstream effects through interactions with an array of effector proteins, including protein kinases, lipid kinases, phospholipases, and various adaptor proteins. The active state of Rho GTPases is terminated by hydrolysis of the bound GTP, a process that is facilitated by GTPase-activating proteins (GAPs; ~70 have been identified to date) [11]. RhoA, Rac1, and Cdc42 are the most extensively characterized members of the Rho protein family. RhoA regulates the formation of contractile actin/myosin stress fibers and the organization of focal contacts [21], Rac is involved in the formation of lamellipodia and focal complexes [21], and Cdc42 governs the formation of filopodia or microspikes as well as focal contacts [22]. Accordingly, these GTPases have crucial roles in several cellular processes, including morphogenesis, migration, cytokinesis, phagocytosis, and cell-matrix contacts [23]. However, it is the role of Rho GTPases in regulating the actin cytoskeleton that is particularly important during a bacterial infection process [11].

Bacterial pathogens do not usually interact directly with actin filaments themselves. But, they subvert and control the polymerization of actin filaments by modulating cellular regulators of this process [5]. Actin interacts with a variety of proteins. Around 150 different known specific actin-binding proteins (ABPs) are found both at extracellular and intracellular (mainly) localizations that modify particular properties or its supramolecular organization [6]. The ABPs can be grouped into at least eight classes: (a) proteins that stabilize or sequester the monomeric actin; (b) proteins that bind along F-actin filaments (such as tropomyosin); (c) motor proteins that generate the force for the sliding of F-actin filaments; (d) proteins that nucleate actin polymerization; (e) proteins that bundle F-actin filaments; (f) proteins that stabilize filament networks; (g) proteins that cut F-actin filaments; (h) proteins that attach filaments to specialized membrane areas [24]. Even if they have different functions many of these proteins attach to a few target zones on the actin surface such as the hydrophobic region. Maybe because of these multiple interactions, the sequence and three-dimensional structure of actin has been very conserved during the billions of years of evolution. Many ABPs are at the end of signaling cascades and are regulated by phospholipid interaction, Ca^2+^-ion concentration, phosphorylation, or small GTPases [25]. These signals either deactivate or activate the supramolecular organization of actin during cell migration, exocytosis or endocytosis, or cytokinesis [8].

Thus, the actin cytoskeleton is a dynamic structure necessary for cell and tissue organization, including the maintenance of epithelial barriers. The epithelial barrier regulates the movement of ions, macromolecules, immune cells, and pathogens, and it is thus essential for normal organ function. Disruption in the epithelial barrier has been shown to coincide with alterations of the actin cytoskeleton in several disease states. These disruptions primarily manifest as increased movement through the paracellular space, which is normally regulated by tight junctions (TJ) [26]. The TJs restrict the movement of pathogens and large macromolecules through the space between two cells [27]. Disruption of the junctions, and the barrier they establish, is a common feature of disease states and is associated with the establishment of infections, increased inflammation, and malabsorption [28]. The actin cytoskeleton is directly connected to cell junctions and plays an important role in the assembly and maintenance of these structures [29].

The main barrier function of the epithelium is thought to depend on tight junctions, which are connected with the actin cytoskeleton. The TJ is closely associated with the actin cytoskeleton and even subtle modulation of the cortical actin cytoskeleton can induce structural changes to the TJ. This strong association is attributed to the direct contacts between several TJ-localized protein components and actin [29]. Three types of transmembrane proteins are part of tight junctions: occludin, claudins, and junctional adhesion molecules (JAMs), and they are connected to adaptor proteins such as zonula occludens 1 (ZO-1), ZO-2, and ZO-3 [11]. Claudins are necessary for the barrier formation, while occludin and JAM family proteins seem to be required for the regulation of barrier permeability and signaling [30]. Many additional proteins are also essential, including PAR6, atypic Ca^2+^- and diacylglycerol-independent protein kinase C (aPKC), and PAR3. This proteins complex, which is important for cell polarity, is regulated by Cdc42, as is the CRUMBS3-PALS1-PATJ-complex, which is essential for tight junction assembly [31]. The precise role of RhoA and Rac in the tight junction regulation has still to be elucidated. Recently, it was suggested that RhoA-dependent phosphorylation of occludin is crucial for the tight junction function [32].

Thus, the F-actin filaments are highly dynamic structures, whose supramolecular organization is constantly modified according to cellular needs. Their dynamic behavior is regulated by a large number of binding proteins that are often the effectors of intracellular and extracellular signaling pathways. It is therefore not surprising that the actin cytoskeleton is one of the main targets of bacterial proteins, and thus of major importance for the host-pathogen interaction [8]. Bacteria have developed numerous toxins and effectors to target the actin cytoskeleton. To induce cytoskeletal changes, pathogenic microbes must ensure delivery of effector molecules onto or into host cells. Bacterial effectors are typically proteins that interface with and influence host-cell pathways and can facilitate the disease. Bacteria use several methods to deliver effector proteins to the host cell. Some effectors, such as toxins, are secreted by bacteria in the vicinity of the host cell, where they bind specific receptors and are taken up by endocytosis [33]. Other effector proteins can facilitate their own uptake by pore-forming subunits or autotransporter domains. Some gram-negative pathogenic bacteria have acquired sophisticated “molecular syringes,” such as type III or type IV secretion systems, which are multisubunit molecular machines that span the bacterial and host membranes and translocate effectors directly into host cells [34, 35].

As mentioned before, the common and recurring target of pathogens is the host cell cytoskeleton, which is utilized by these microbes for purposes that include attachment, entry into cells, movement within and between cells, vacuole formation and remodeling, and avoidance of phagocytosis. A relevant example is *Escherichia coli*, which is a bacterial species genetically versatile that comprises a huge group of nonpathogenic and pathogenic bacteria. *E. coli* is an important member of the normal intestinal microflora of humans and other mammals. Due to its genome plasticity through horizontal transfer, *E. coli* has been widely exploited as a cloning host in recombinant DNA technology. *E. coli*, beside being a laboratory workhorse or harmless intestinal inhabitant, is a highly versatile and frequently deadly pathogen. Several different *E. coli* strains cause diverse intestinal and extraintestinal diseases by means of virulence factors that affect a wide range of cellular processes.

## 3. Diarrheagenic *Escherichia coli*

There are several highly adapted *E. coli* clones that have acquired specific virulence attributes, which confer an increased ability to adapt to new niches and allow them to cause a broad spectrum of disease. The virulence factors are often encoded on genetic elements, that can be mobilized into different strains to generate novel combinations of virulence factors. Only the most successful combinations of virulence factors have persisted to become specific *E. coli* pathotypes that are capable of causing disease in healthy individuals [36]. Among the intestinal pathogens, there are six categories of pathotypes: enteropathogenic *E. coli* (EPEC), enterohemorrhagic *E. coli* (EHEC), enterotoxigenic *E. coli* (ETEC), enteroaggregative *E. coli* (EAEC), enteroinvasive *E. coli* (EIEC), and diffusely adherent *E. coli* (DAEC) [36]. Most of the pathogenic *E. coli* strains remain extracellular, but EIEC is a true intracellular pathogen that is capable of invading and replicating within epithelial cells and macrophages. Other *E. coli* strains might be internalized by epithelial cells at low levels, but do not appear to replicate intracellularly [34].

Thus, *E. coli* pathotypes interact with epithelial cells and these interactions depend on the specific combination of virulence factors that define their life style and their capability to cause a specific pathology (pathotype). Except for ETEC, which produce the heat-labile (LT) and heat-stable (ST) enterotoxins that are their main virulence factors [36], the other five pathotypes subvert and control the polymerization of actin filaments by modulating cellular regulators of this process. EIEC manipulates the cytoskeleton for invasion of a host cell and/or to gain motility in the cell. After invasion and escape from membrane-enclosed vesicles into the cytosol, EIEC also manipulate actin-filament dynamics to move within the infected host cell [4]. They do so by recruiting actin in one of their poles, through bacterial-protein-mediated nucleation of actin. The hijacking of actin-associated cytoskeletal components can also occur during the infection with extracellular pathogens, such as EPEC and EHEC, which share similar features, and DAEC [5]. Finally, virulence factors secreted by EAEC and DAEC, which cause proteolysis of proteins that stabilize actin filament networks.

## 4. Enteroinvasive *E. coli*

**Figure 1:**
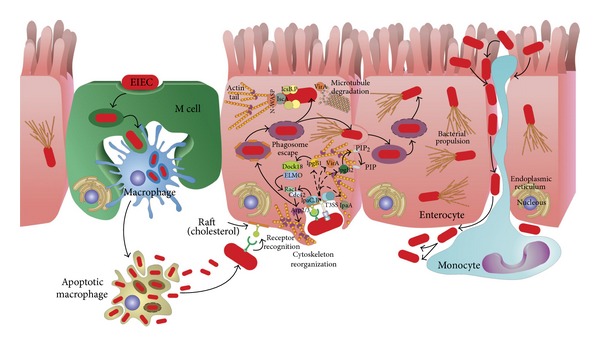
A model for *Shigella*/EIEC infection of colonic epithelial cells. *Shigella*/EIEC invade the epithelium from the intestinal lumen through M-cells. After reaching the epithelium they invade epithelial cells and are phagocytosed by resident macrophages. *Shigella*/EIEC escape the phagosome of both cells but while *Shigella*/EIEC replicate within epithelial cells, they induce apoptosis in macrophages. Bacteria are released and can invade the epithelial cells from the basolateral side, move into the cytoplasm by triggering actin polymerization, and spread to adjacent cells (for full details, see main text).

Enteroinvasive *Escherichia coli* (EIEC) are closely related to *Shigella* spp., showing remarkable phenotypic and genotypic similarities. However, the disease induced by EIEC is generally less severe than that induced by *Shigella* spp. [37]. This difference in severity may be associated with a lower expression level of virulence factors by EIEC in the presence of various host cells, such as macrophages and intestinal epithelial cells. Phylogenetic studies have suggested that *Shigella* and EIEC form a single pathovar of *E. coli*. EIEC strains are regarded as precursors of full-blown *Shigella* evolved from *E. coli* [38]. *Shigella flexneri* and EIEC are pathogenic microorganisms known to cause disease in humans by very similar mechanisms of pathogenicity. Once ingested, bacteria reach the colonic mucosa and invade various cell types, including M cells, macrophages, and epithelial cells. Invasion of the intestinal mucosa is followed by intracellular bacterial multiplication, spread of the infection to adjacent cells, induction of severe inflammation of the colon, and destruction of the mucosa [39]. The virulence of these strains depends on the presence of both chromosomal genes and a large virulence plasmid (pINV). pINVs represent a family of highly related plasmids, and they are functionally interchangeable between *Shigella* and EIEC strains. Several virulence traits (Figure 1) are encoded by pINV genes, including (i) the ability to invade and multiply within epithelial cells [40], and (ii) the ability to spread infection intracellularly and to adjacent cells [41].

The uptake of *S*. *flexneri*/EIEC depends on complex rearrangements of membranes and the actin cytoskeleton, which are mediated by the controlled temporal and spatial actions of a multitude of bacterial and host cell factors [42]. The bacteria directly target intracellular host cell signaling pathways by transporting effector proteins across the bacterial inner membrane (IM) and outer membrane (OM) as well as the host plasma membrane. The *S. flexneri*/EIEC Mxi-Spa T3SS is a representative of sophisticated molecular export machineries known as injectisomes, that translocate proteins into the host cell cytoplasm in one step [43]. The initial contact between *Shigella*/EIEC and host cells takes place at lipid raft membrane domains and is mediated by the receptors CD44 and *α*5*β*1 integrin. Receptor binding induces early actin cytoskeleton rearrangements and primes the cell for uptake, but the efficient and complete engulfment of the bacteria is triggered by the translocation of at least six T3SS effector proteins. IpaB, IpaC, and IpaD elicit actin rearrangements at the site of bacterial attachment that are required for entry, whereas IpaA promotes the reorganization of these actin-rich structures [44]. IpaB binds to CD44, a member of the immunoglobulin superfamily that binds hyaluronan. This interaction is required for *Shigella*-induced cytoskeletal rearrangements that lead to bacterial entry, presumably by determining early events of the internalization process [45].

The reorganization of the eukaryotic cytoskeleton is governed predominantly by small Rho GTPases and tyrosine kinases, which renders these enzymes targets for manipulation by pathogenic bacteria [46]. *S. flexneri*, as well as many other bacterial pathogens, requires and manipulates these Rho GTPase pathways to promote its uptake [46]. *S. flexneri* induces massive actin polymerization, leading to the formation of large membrane protrusions at the entry site, which form a macropinocytic pocket enclosing the bacteria. This process requires the activation of the small GTPases Rac1 and Cdc42, that recruit the actin-nucleating complex Arp2/3 [47]. Several bacterial effectors have been implicated in the induction of actin polymerization. The translocator protein IpaC is able to initiate actin nucleation and the formation of filopodial and lamellipodial membrane extensions, but it is unclear how IpaC stimulates Rac1 and Cdc42 [47]. IpaC invasion function requires its immediate C-terminus and this general region may be involved in its ability to trigger actin nucleation. A pivotal role for the T3SS effector IpgB1 in triggering invasion has been suggested [48]. IpgB1 mimics the active small GTPase RhoG and elicits Rac1 activation and membrane ruffling through the ELMO-Dock180 pathway [49]. Rac1 is further activated in response to the destabilization of the microtubule network by the *S. flexneri* VirA, a cysteine protease [50]. Mxi-Spa effectors, which induce actin polymerization at the entry site, might also include IpgB2. IpgB2 (a IpgB1 homologue) was shown to mimic the GTP-bound form of RhoA, and its expression in eukaryotic cells triggered the formation of actin stress fibers and membrane ruffling [51]. However, the specific contribution of IpgB2 to the signaling induced in the course of an infection of epithelial cells with *S. flexneri* remains to be established. Two additional T3SS effector proteins are necessary for the full invasiveness of *S. flexneri*. The phosphoinositide 4-phosphatase IpgD hydrolyzes phosphatidylinositol-4,5-biphosphate [PtdIns(4,5)P2] to yield phosphatidylinositol-5-phosphate [PtdIns(5)P] [52]. Hydrolysis of [PtdIns(4,5)P2] leads to the dissociation of the actin cytoskeleton from the plasma membrane, which facilitates the remodeling of membranes and actin. Finally, the translocated effector IpaA binds vinculin and enhances its association to actin filaments, thus mediating localized actin depolymerization and a reduction of the adhesion between cells and the extracellular matrix. This process might promote the closure of the phagocytic cup around the bacteria [53].

After triggering its uptake into host cells, *S. flexneri*/EIEC is entrapped in the phagosome. After that, *S. flexneri* lyses the surrounding membranes and escapes into the cytoplasm in less than 10 min [54]. Membrane lysis does not depend on the acidification of the phagosome and is mediated by the Mxi-Spa T3SS and the translocator proteins IpaB, IpaC, and IpaD [55]. Although all three translocator proteins are needed for the escape from the phagosome, there is evidence that IpaC is the decisive factor mediating membrane lysis.

The cytoplasm of epithelial cells is the main replicative niche for *S. flexneri*/EIEC. The intracellular survival and intercellular spread of *S. flexneri* are linked to the machinery allowing the bacteria to move by directed actin polymerization. The molecular mechanisms mediating the actin-based motility of *S. flexneri* and other intracellular pathogens have been studied extensively [4]. IcsA (intracellular spread) protein, which localizes to one pole of the bacterium, is the central bacterial mediator of actin polymerization [56, 57]. Surface-bound IcsA recruits and activates host cell factors, including N-WASP and the Arp2/3 complex [58]. The complex thus formed serves as an actin nucleator and catalyzes the directed elongation of an actin tail, which propels *S. flexneri*/EIEC through the cytoplasm. In addition to the actin-nucleating complex, the T3SS-secreted cysteine protease VirA was recently identified to be pivotal for efficient intracellular movement and subsequent intercellular spread [59]. VirA degrades *α*-tubulin, thus creating a “tunnel” in the dense intracellular microtubule network. However, a large body of evidence, including structural studies, has now shown that VirA is not a cysteine protease and does not directly destabilize microtubules [60, 61]. Intracellular motility, replication, and survival further depend on the T3SS substrate IcsB, that protects the bacteria from being recognized and entrapped by the host cell autophagy machinery [62]. Autophagy is a host cell recycling and defense system, which engulfs and sequesters cytoplasmic components in double-membrane-bound compartments [63]. Interestingly, IcsA contains an autophagy-inducing recognition site, which has to be masked by IcsB to prevent engulfment by autophagic vacuoles and to ensure the intracellular survival of *S*. *flexneri* [62]. Due to propulsion through the host cell cytoplasm by actin polymerization, moving *S. flexneri* eventually impinges on the plasma membrane at the tight junctions of adjacent epithelial cells. The arising protrusions can be endocytosed by the neighboring cell in a process requiring host cell factors like myosin light-chain kinase [64]. After lysis of the surrounding double membrane, which depends on the T3SS and the translocator proteins IpaB, IpaC, and IpaD, *S. flexneri* is free in the cytoplasm, and a new cycle of replication and cell-to-cell spread can start [55, 65].

## 5. Enteropathogenic and Enterohemorrhagic *E. coli*

Enterohemorrhagic *E. coli* (EHEC) and enteropathogenic *E. coli* (EPEC) cause serious diarrhea in humans that can result in death [36]. EPEC, EHEC, and a number of animal pathogens such as the mouse-specific pathogen *Citrobacter rodentium* and rabbit-specific enteropathogenic *E. coli* (REPEC) [66–68] belong to a group of mainly extracellular diarrheagenic pathogens that colonize the gut epithelium by attaching and effacing (A/E) lesions [69]. The A/E histopathology is characterized by effacement of the brush border microvilli, intimate bacterial adherence to the enterocyte apical plasma membrane, and the accumulation of polymerized actin beneath the attached bacteria [70]. All A/E pathogens carry a pathogenicity island, the locus of enterocyte effacement (LEE) [71] that encodes gene regulators [72, 73], the adhesin called intimin [74], a type III secretion system (T3SS) [71], chaperones [75, 76], and several secreted proteins, including the translocated intimin receptor called Tir [76, 77].

**Figure 2:**
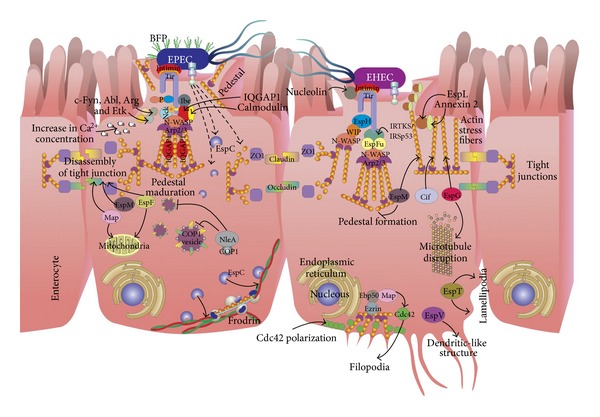
Models of actin cytoskeleton manipulation by enteropathogenic *E. coli* (EPEC) and enterohemorrhagic *E. coli* (EHEC). EPEC and EHEC are attaching and effacing (A/E) pathogens that destroy the microvilli and subvert host cell actin to form pedestals beneath the attachment site. EPEC attaches to the small bowel through the bundle-forming pilus (BFP), forming localized adhesions. Using the type III secretion system, a large repertoire of effector proteins is injected into the host cell. Intimate attachment is mediated by the interaction between the translocated intimin receptor (Tir) and intimin, an adhesin. Tir is phosphorylated by host tyrosine kinases, and phosphorylated Tir recruits Nck, which activates neural Wiskott-Aldrich syndrome protein (N-WASP) and the actin-related protein 2/3 (Arp2/3) complex to mediate actin rearrangements and pedestal formation. Whereas, the mechanism of pedestal formation by EHEC is slightly different from that used by EPEC. Tir is not phosphorylated, and pedestal formation is Nck-independent, but it is mediated by Tir cytoskeleton-coupling protein (EspF_u_; also known as TccP), which is linked to Tir through the host protein insulin receptor tyrosine kinase substrate (IRTKS) and interacts with N-WASP to activate the Arp2/3 complex. EHEC injects many of the same effectors as EPEC into the host cell to manipulate host processes (for full details, see main text).

EPEC is a cause of gastroenteritis in infants less than 2 years of age, while EHEC causes bloody diarrhea in children and the elderly. EHEC is distinguished from EPEC by the production of Shiga toxins that can cause severe kidney damage leading to the hemolytic uremic syndrome, a form of acute renal failure [78]. Upon contact with epithelial cells, EHEC/EPEC inject a variety of effectors into the cells to modulate cellular functions involved in the host defense response, the dynamics of cytoskeleton and the maintenance of tight junctions (Figure 2). A major target of virulence factors is the cellular signaling cascade involved in the construction and modulation of the cytoskeleton and microfilaments. While the bacteria remain mostly extracellular in the lumen of the gut, the T3SS effectors of A/E pathogens access and manipulate the intracellular environment of host cells. The effectors subvert various host cell processes, which enable the bacteria to colonize, multiply and cause the disease. A group of 21 core effectors, including all those encoded within LEE and a number of non-LEE effectors, are shared by all A/E pathogens [79, 80].

The T3SS is an injection device (injectisome) that can transfer bacterial virulence proteins directly into host cells. The apparatus is made up of a basal body that spans both bacterial membranes and an extracellular needle that possesses a channel that is thought to act as a conduit for protein secretion [81]. The needle projects from the basal body and is assembled by the polymerization of a single, small needle subunit [82]. Contact with a host-cell membrane triggers the insertion of a pore into the target membrane, and effectors are translocated through this pore into the host cell. The T3SS exports two distinct categories of proteins: the translocators that form a pore in the target membrane and the effectors that traffic through this pore into the host cell.

LEE encodes the translocator proteins EspA, EspD, and EspB, which are part of the T3SS and are required for the translocation of effectors into the host cell. Other factors encodes by LEE include the adhesin intimin, which binds to Tir and is essential for the intimate attachment of the bacteria with the cytoplasmic membrane of the host cell. Other effector proteins such as EspF, EspG, EspZ, EspH, Map, and Tir, some chaperone proteins such as CesD for EspB and EspD, CesF for EspF, and CesT for Tir are also encoded within LEE [83].

### 5.1. Tir

One of the most important effectors studied that manipulates the actin cytoskeleton by EHEC-EPEC is translocated intimin receptor (Tir). EHEC-EPEC Tir mainly helps the bacteria to bind intimately to the epithelial cell surface. This intimate union requires intimin in the outer membrane of the bacterium and Tir embedded into the epithelial cell membrane. In sites of contact with epithelial cells, EPEC Tir is translocated via T3SS and is inserted into the plasma membrane of epithelial cells, where it is tyrosine phosphorylated and acts as a receptor for intimin [84]. This effector is considered the most important in the process of manipulating the actin cytoskeleton and the formation of actin-rich structures known as pedestals. Among the Tir C-terminal sequence, there are regions necessary to carry out the processes of controlling the actin machinery [85, 86]. Although EPEC Tir (Tir_EPEC_) and EHEC Tir (Tir_EHEC_) are structurally identical (60–67% identity) [84], the signaling pathways to manipulate the actin cytoskeleton within the epithelial cell are very different.

In the case of Tir_EPEC_, Tir has two transmembrane domains and six tyrosine residues, all located in the C-terminus. A loop model has been proposed for Tir when is inserted into the host membrane, where it uses its two transmembrane domains to traverse the membrane of the enterocyte, with their N- and C-terminal regions located inside the host cell; the region between the two transmembrane domains form a extracellular hairpin. Tir-intimin interaction guides the intimate attachment of EPEC to epithelial cells, leading to the pedestal formation and actin polymerization [77]. Tir_EHEC_ and Tir_EPEC_ show a high degree of amino acid identity, particularly in their N-termini but are most divergent in their N-terminal domains. This region of the protein contains tyrosine residues that in Tir_EPEC_ are potential substrates for phosphorylation. Tir_EHEC_, which is not tyrosine phosphorylated, lacks one of these residues [87]. Thereby, pedestal formation by EHEC and EPEC differs in the requirements for Tir tyrosine phosphorylation and additional bacterial factors delivered to the host cell. Tir_EPEC_ is phosphorylated by the host cell kinase, c-Fyn, just after it is translocated to the epithelial cell membrane. Subsequently, Abl, Arg, and Etk through their SH3 domains recognize the proline-rich motif of Tir at the N-terminus then maintain the tyrosine phosphorylation persistent and turning the signaling pathway on to polymerize actin under attached bacteria, on the pedestal [88, 89]. In Tir_EPEC_, a 12-residue peptide encompassing phosphorylated Y474 binds the SH2 domains of the Nck adaptor proteins. WIP-like proteins may be involved in the subsequent recruitment of N-WASP to a complex of Tir and Nck, but the three tandem SH3 domains of Nck1 and Nck2 likely activate N-WASP by directly binding to its PRD [90]. Multimerization of N-WASP can further enhance Arp2/3-mediated actin nucleation and pedestal assembly, which causes membrane protrusion beneath the bacterium [91]. It worthy to note that actin pedestal formation observed *in vitro* is different to A/E lesion formation *in vivo*, as both tyrosine residues are not required for A/E lesion formation, demonstrating that *in vitro* phenotypes do not always correlate with that of *in vivo* observations [92].

There are other proteins that bind to Tir and modulate the actin cytoskeleton, such as IQGAP1. IQGAP1 is involved in diverse cellular functions, since GTPase signaling to control cell proliferation and motility. IQGAP1 binds directly to actin, promoting the polymerization and placing it onto actin lamellipodia. It has been determined that certain middle region of the IQGAP1 C-terminal binds to the CRIB domain of N-WASP, removing autoinhibition of the latter and promoting actin polymerization, such as Cdc42 does [93]. IQGAP1 binds to Tir, causing IQGAP1 accumulation at sites of adherent bacterial. Since the Ca^2+^ signaling is required for remodeling and handling polymerization actin machinery in the cell surface, an increase in the Ca^2+^ concentration is required for EHEC-EPEC pedestal formation. The increase in Ca^2+^ intracellular concentration promotes the association of IQGAP1 with calmodulin, recruiting the last to the pedestal. This interaction reduces the interaction of IQGAP1 with Rac1 and Cdc42. The recruitment of IQGAP1 to the pedestal increases the activation of N-WASP and therefore the actin polymerization and formation of pedestal in EHEC-EPEC adherent sites [94].

Another T3SS effector, termed IQGAP1-binding effector protein (Ibe) was recently discovered to be associated with LEE. This effector was shown to regulate Tir phosphorylation and actin pedestal formation [94]. Like Tir, IQGAP1 binds Ibe and colocalizes with Ibe at actin pedestals. However, the mechanism underlying the contribution of Ibe to EPEC pathogenesis remains to be defined [95]; it should also be noted that Ibe is not conserved and thus not a core LEE efector.

EHEC and EPEC have evolved somewhat different mechanisms to activate the actin polymerization machinery. EHEC also generates pedestals in a Tir dependent manner without recruitment of Nck to the sites of adherent bacteria, but in the absence of tyrosine phosphorylation, EHEC translocates a second effector protein, EspFu [96]. Although EHEC does not recruit Nck, N-WASP is recruited and necessary for the pedestals formation by EHEC [97]. The expression of EspFu in EHEC allows the same adaptor to function independently of tyrosine phosphorylation. All the information necessary to manipulate the actin polymerization signaling pathway lay in a 12 amino acids region at the C-terminal (452-463) of Tir_EHEC_ [98]. This region contains a tyrosine (Y458), which is not phosphorylated, but is very important in the formation of a motif known as NPY_458_. This motif is also crucial for the process of actin polymerization by EPEC independently of Nck, which uses the NPY_454_ motif in the C-terminal of Tir_EPEC_ [99]; although the T3SS effector translocated by EPEC could be related to NPY_454_ the signaling pathway has not yet been discovered. The NPY_458_ motif at the C-terminal of Tir_EHEC_ is detected by two homologous eukaryotic proteins that are members of the I-BAR (Bin-Amphiphysin-Rvs167) family, IRTKS, and IRSp53. These proteins belong to the I-BAR family because its amino acid sequence laids I-BAR domains that bind to the eukaryotic membrane deforming it and producing bumps. These proteins also have SH3 domains, which help them to recognize proline-rich motifs [100]. IRTKS and IRSp53 contain an IMD (IRSp53/MIM-homology) domain within its N-terminal sequence, which normally deforms the eukaryotic membrane and binds to eukaryotic small G proteins [100]. Through its IMD domain, IRSp53 and IRTKS, recognizes the NPY_458_ motif of Tir_EHEC_, while IRTKS and IRSp53 use the SH3 domain to recognize the repetitive proline-rich protein EspFu, which is also translocated by EHEC T3SS. Then EspFu activates N-WASP, allowing a potent activation of actin polymerization mediated by N-WASP/Arp2/3 [86]. Recently, it was found that spectrin is a key protein involved in the signaling required for actin polymerization during EHEC pedestal formation. A colocalization of spectrin cytoskeletal proteins with IRSp53 and IRTKS, which are required for EHEC pedestal formation, has also been reported [101].

EspFu, also known as TccP (Tir cytoskeleton-coupling Protein) is an effector protein that is translocated by the EHEC T3SS. This effector is encoded on a cryptic phage known as 933U, outside the pathogenicity island LEE. EspFu comprises a translocation signal in the N-terminus recognized by the T3SS, followed by five and a half 47 amino acid repeats each, which are rich in prolines. Within each of these repeats, the first 17 amino acids are important for activation of N-WASP, which is normally given by other molecules such as the small GTPases Cdc42, the adaptor protein Nck and phosphoinositides such as PtdIns(4,5)P2 [102]. EspFu controls actin by activating members of the WASP family. EspFu binds to the autoinhibitory GTPase binding domain (GBD) in WASP proteins displacing it from the activity-bearing VCA domain (for verprolin homology, central hydrophobic, and acidic regions). This interaction potently activates WASP and neural (N)-WASP *in vitro* and induces localized actin assembly in cells. EspFu forms an amphipathic helix that binds the GBD, mimicking interactions of the VCA domain in autoinhibited WASP. Thus, EspFu activates WASP by competing directly for the VCA binding site on the GBD [103]. The *α*-helix of 17 amino acid binds to the autoinhibitory domain GBD (GTPase-binding domain) of N-WASP, releasing the catalytic domain WCA (WASP homology C-terminal acidic domain 2 connector), which activates the Arp2/3 complex [103, 104]. Thus, EspFu repeats cooperate to promote synergistic activation of N-WASP and Arp2/3-mediated actin assembly in EHEC adherent sites [102].

### 5.2. Other LEE Encoded Effectors

*EspF* is one of many effector proteins exclusive to the A/E pathogen that includes EPEC and EHEC. EspF is one of the most known multifunctional effector proteins, with roles in several host cellular processes, including disruption of the epithelial barrier, antiphagocytosis, microvillus effacement, host membrane remodeling, modulation of the cytoskeleton, targeting and disruption of the nucleolus, intermediate filament disruption, cell invasion, mitochondrial dysfunction, apoptosis, and inhibition of several important epithelial transporters. For the purpose of this paper, we will only highlight those functions related or associated to the actin cytoskeleton.

EspF exhibits a modular architecture composed of several distinct functional domains including a well-conserved N-terminal region (residues 1 to 20) containing the bacterial secretion signal that is sufficient for EspF secretion and translocation into host cells [105]. In the same N-terminal region are two organelle-targeting domains; one superimposed with the secretion signal that directs EspF to the host mitochondria (residues 1 to 24) and another to the nucleolus (residues 21 to 74) [81, 106]. The remaining C-terminal region of EspF (residue 74 and onward in all variants) comprises the eukaryotic-like proline-rich repeats (PRR), which are almost identical in size and sequence among the EspF variants [107, 108]. Each PRR comprises two putative overlapping Src homology 3 (SH3) binding domains with the consensus PxxP motif that is reported to mediate the binding to a large number of eukaryotic signaling proteins containing the well-characterized SH3 domain [109]. The PRR modules also contain a functional N-WASP binding motif [107] toward the C-terminal end of each repeat. EspF has been found in all A/E pathogens, although its size varies, being dictated by the number of PRR modules, with the rabbit-specific EPEC strain RDEC-1 possessing two, prototypical EPEC three, EHEC four, and *C. rodentium* five modules [107, 108]. Some A/E pathogens carry additional EspF homologues, such as Tccp/EspF(U), which displays partial similarity to EspF and possesses the PRR regions [85, 110]. However, Tccp/EspFu effectors, as mentioned before, play a clear role in EHEC manipulation of the cytoskeleton.

EspF has been found to bind several host proteins including actin, profilin, Arp2, N-WASP, sorting nexin 9 (SNX9), Abcf2, and the intermediate filament protein cytokeratin 18 [107, 108, 111, 112]. EPEC EspF has six putative PxxP SH3 binding motifs within its three PRR domains, and two studies have revealed that it specifically binds to the SH3 domain of the host protein SNX9. EspF interacts with SNX9 in the cytosol during the infection to induce the formation of membrane tubules [107, 113]. The preferred EspF binding site of SNX9 is RxAPxxP, a repeat sequence found at the N-terminal end of each PRR module [107]. In the host cell, SNX9 is normally involved in host membrane cytoskeleton processes, and the binding of EspF to SNX9 appears to regulate host membrane alterations [107]. However, the role of membrane remodeling during bacterial infection has not been determined and it is unlinked to the disruption of tight junctions (TJ), NHE3 inhibition (see below), or antiphagocytosis [114]. The putative N-WASP binding motif of each PRR has also been shown to be functional, mediating the direct interaction of EspF with the Cdc42/Rac-interactive binding (CRIB) domain of N-WASP [107]. All EspF variants possess the N-WASP binding sites, suggesting some level of functional similarity between these effectors. *In vitro* biochemical studies have revealed that EspF binds and activates N-WASP, which subsequently induces actin polymerization [107]. Interestingly, a recent study has shown a direct interaction between actin itself and EspF from rabbit EPEC strain E22 [108]. In the same study, EspF was shown to contribute to the extent and size of actin-based pedestals in EPEC-infected cells, presumably through its direct modulation of the cytoskeleton, although no role for N-WASP was demonstrated [108]. EspF modulation of actin may be linked to several prominent host cellular changes, including microvillus elongation [115], microvillus effacement [116], TJ disruption [117], and antiphagocytosis [118]. Therefore, the ability of EspF to bind actin and signal through N-WASP may have important implications in the disease [108]. Additional to its role on the actin cytoskeleton, EspF also binds cytokeratin 18, a protein that forms part of the intermediate filament network in epithelial cells, and this interaction was suggested to facilitate the collapse of this important cellular structure [112].

### 5.3. Map

Mitochondrial associated protein (Map) was first described as an EPEC effector protein that is targeted to the mitochondria [119] via a small peptide signal corresponding to the N-terminal 44 amino acids. Map alters mitochondrial morphology and membrane potential *in vitro* and *in vivo* [120]. In addition, Map triggers transient formation of filopodia in cultured human cell lines [51, 121]. Map activates Cdc42 [121], but it has also been suggested that Map can bypass Cdc42, triggering filopodia via a Cdc42 molecular mimicry mechanism [51]. Moreover, Map was shown to bind, via its C-terminal PDZ ligand motif TRL, PDZ1 of the scaffold protein sodium/hydrogen exchanger regulatory factor-1 (NHERF1) [51]. However, structural studies of Map have demonstrated Map mimics the activation mechanism of RhoGEFs and is not a Cdc42 mimic as previous postulated [122].

Berger et al. [123] have suggested a model in which EPEC infection triggers signal transduction pathways that while individually lead to either filopodia or pedestal formation, when crossed over fine-tune modulation of actin dynamic for the benefit of the adherent bacteria. According to this model, Map activates Cdc42, either directly or via the interaction with as yet unidentified GEF, leading to the filopodia formation. Before clearing the cytosol and targeting the mitochondria, Map (alone or in complex with the GEF) transitorily interacts via its PDZ ligand motif with PDZ1 of NHERF1 [51, 124]. This leads to recruitment of activated ezrin [125]. Ezrin can then interact with GEF Dbl [126] or with Rho guanine nucleotide dissociation inhibitors [127], leading to activation of RhoA [127] and the RhoA-ROCK pathway, which stabilizes the actin microfilaments within the filopodia via phosphorylation of cofilin [128].

Activation of Tir signal transduction pathway (see above), probably in conjunction with Map mitochondria targeting, signals filopodia withdrawal. Filopodia downregulation could be due to the fact that Nck sequesters N-WASP from the Cdc42-GTP pathway, since Nck has a higher affinity to N-WASP [129]. Alternatively, Tir-bound Nck might trigger local activation of GC-GAPs [130], which were reported to specifically inactivate Rac-1 and Cdc42. It has been suggested that filopodia withdrawal from infected HeLa cells is due to a GAP “GXLR” motif localized at the C-terminus of Tir [121].

Recently, Orchard et al. [131] assess the effects of Map on F-actin dynamics and Cdc42 activation and found that, in the absence of external cues, Map polarized Cdc42 on the cell surface, resulting in the formation of spatially restricted clusters of actin-rich membrane protrusions. In addition to its GEF domain, Map contains a C-terminal PDZ domain-binding motif that interacts with EBP50, and both domains are required to polarize Cdc42 activity on the cell membrane. Analysis of the Map-Cdc42 signaling cascade showed that EBP50 does not target Map to cell membrane receptors. In its place, EBP50 formed a scaffolding complex with another host cell protein, ezrin, which linked Map to the actin cytoskeleton. *In vitro* analysis, combined with structural and mathematical modeling of the minimal MapABD (fusion of the actin-binding domain (ABD) of ezrin directly to Map) signaling network, revealed a key role for actin dynamics in the localization of Map and Cdc42. The models suggested that Cdc42 activation occurs as a result of the stochastic cycling of Map and F-actin between the cytosol and the plasma membrane, and that actin nucleation locally amplifies Cdc42 signal transduction through a Map-dependent positive feedback loop [132].

### 5.4. EspG

EspG and EspG2, conserved proteins of A/E pathogens, including EPEC, EHEC and *C. rodentium* [115], induce the disruption of microtubule networks beneath adherent bacteria by the direct association of EspG and EspG2 with tubulins [115]. EspG2 is a distant, redundant homolog of LEE-encoded EspG that is not LEE-encoded [115]. Both EspG and EspG2 effectors share homology with the *Shigella* VirA effector. Although it has been demonstrated that *Shigella* VirA promotes the destabilization of host microtubules and Rac1 activation [50], the contributions of EspG/EspG2 effectors to EPEC virulence remain unclear. Moreover, EspG and EspG2 effectors are involved in the induction of actin stress fiber formation in a Tir-intimin interaction-independent manner [133]. Interestingly, GEF-H1 (a guanine nucleotide exchange factor) is exploited by EspG/EspG2 effectors to induce actin stress fiber formation during EPEC infection. GEF-H1 switches to the active form as a result of its dissociation from microtubule networks. Activated GEF-H1 promotes the binding of GTP to RhoA, resulting in the activation of RhoA. Finally, the activation of ROCK, which is located downstream of the RhoA signal, induces actin stress fiber assembly and probably exerts an influence on paracellular permeability in EPEC-infected cells [134]. Yeast expressing EspG were unable to control cortical actin polarity [133]. Furthermore, while the deletion of *espG* alone had no effect on the decrease in TER by EPEC, deletion of both *espG* and *espG2* significantly delayed TJ disruption of T84 monolayers as measured by TER [135]. Recently, it has been found that EspG binds directly to the N-terminal inhibitory domain of human p21 activated kinase (PAK), which binds GTP-bound Rac or Cdc42. EspG directly binds to the inhibitory domain of PAK, mimicking a small GTPase, implying that the primary role of EspG during pathogenesis is to promote actin remodeling [136]. EspG binds to the Rac/Cdc42-binding site of PAK1 leading to the conclusion that by imitating a small GTPase, EspG permits EPEC to bypass host cell GTPases and enable PAK-dependent actin remodeling regardless of the status of native Rac/Cdc42 [137].

### 5.5. EspH

The effector EspH is efficiently translocated into the infected cells by the T3SS and is localized beneath the EPEC microcolonies. Interestingly, EspH represses the formation of filopodia and enhances the formation of actin pedestals. Moreover, overexpression of EspH by EHEC induces a marked elongation of the typically flat pedestals. The repression of filopodium formation by EspH is independent of Tir. EspH transiently expressed by COS cells was localized to the membrane and disrupted the actin cytoskeletal structure indicating that EspH is a modulator of the host actin cytoskeleton structure [138]. Furthermore, EspH markedly disrupts actin cytoskeleton structure and induces cell rounding up when ectopically expressed or delivered into HeLa cells by the bacterial T3SS. EspH inactivates host Rho GTPase signaling pathway at the level of RhoGEF. Since EspH directly binds to the tandem Dbl-homology and pleckstrin-homology (DH-PH) domain in multiple RhoGEFs, which prevents their binding to Rho and thereby inhibits nucleotide exchange-mediated Rho activation and actin cytoskeleton dynamics [139]. Interestingly, bacterial RhoGEFs are resistant to the DH-PH mammalian RhoGEF inhibitor EspH. Therefore, EPEC and EHEC neutralize mammalian RhoGEFs while translocating their own bacterial RhoGEFs to hijack Rho GTPase signaling for benefiting the pathogen. Moreover, EspH progressively induces disassembly of focal adhesions concomitantly with actin disruption [140]. Recently, it has been found that EspH promotes actin polymerization at the bacterial attachment sites independently of the Tir tyrosine residues Y474 and Y454, which are implicated in binding Nck and IRSp53/IRTKS, respectively. Furthermore, EspH promotes recruitment of N-WASP and the Arp2/3 complex to the bacterial attachment site, in a mechanism involving the C-terminus of Tir and the WH1 domain of N-WASP. Tir and EspH recruit WIP in an N-WASP dependent manner. WIP in EPEC mediates actin polymerization and pedestal elongation and represents the first instance whereby N-WASP is efficiently recruited to the EPEC attachment sites independently of the Tir:Nck and Tir:IRTKS/IRSp53 pathways. These events reveal the intricacies of Tir and EspH-mediated actin signaling pathways that comprise distinct, convergent, and synergistic signaling cascades [141].

### 5.6. EspM and EspT

A number of known T3SS from *Shigella* (IpgB1 and IpgB2), *Salmonella* (SifA and SifB), and EPEC and EHEC (Map) have been grouped together as WxxxE effectors, based on a conserved motif comprising an invariant tryptophan (W) and a glutamic acid (E) separated by three variable amino acids [51]. Recently, new WxxxE effectors encoded by EPEC and EHEC, EspM [142] and EspT [143] were identified. Both proteins are non-LEE encoded effectors. Ectopic expression of Map leads to filopodia formation [51], IpgB2 and EspM trigger stress fibers [51, 144] and IpgB1 and EspT induce membrane ruffles and lamellipodia [51, 145]. These phenotypes are typically associated with activated Cdc42, RhoA and Rac1 [146], respectively. Alto et al. [51] suggested that the WxxxE effectors, which play important roles in cell invasion (IpgB proteins) and intracellular survival (SifA), mimic the function of Rho GTPases. Handa et al. [49] subsequently demonstrated that IpgB1 stimulates formation of membrane ruffles by activating Rac1 through recruitment of the Rac1-specific ELMO-Dock180 GEF complex. Moreover, the structure of SifA in complex with the PH domain of SKIP has shown that its C-terminus domain, which includes the WxxxE motif, adopts a fold similar to SopE [147]. However, neither direct binding to the Rho GTPases nor GEF activity was detected in this study. Furthermore, it has been reported that Map [123], EspM [144], and EspT [145] activate the Rho GTPases Cdc42, RhoA, and Rac1. Recently, Simovitch et al. [148] confirmed that EHEC EspM1 and EspM2 activate the RhoA signaling pathway and induce the formation of stress fibers upon infection of host cells. In addition, they found that EspM inhibits the formation of actin pedestals, and that the translocation of EspM into polarized epithelial cells induces dramatic changes in the TJ localization and in the morphology and architecture of infected polarized monolayers. Surprisingly, despite the dramatic changes in their architecture, cells remain alive and the epithelial monolayer maintains a normal barrier function. All these results show that the EspM effectors inhibit pedestal formation and induce TJ mislocalization, as well as dramatic changes in the architecture of the polarized monolayer [148]. Recently, it has also been shown that EspM2 is a RhoA guanine nucleotide exchange factor. A direct interaction between EspM2 or SifA and nucleotide-free RhoA was identified. EspM2 has a similar fold to SifA and the guanine nucleotide exchange factor (GEF) effector SopE. EspM2 induced nucleotide exchange in RhoA but not in Rac1 or H-Ras, while SifA induced nucleotide exchange in none of them. Substitution of Q124, located within the catalytic loop of EspM2, by alanine, greatly attenuated the RhoA GEF activity *in vitro* and the ability of EspM2 to induce stress fibers upon ectopic expression [149].

Thus, Map and EspM exhibit GEF activity towards Cdc42 [150] and RhoA [149], respectively, thereby triggering formation of filopodia (Map) and stress fibres (EspM) [51, 143, 144]. EspT activates both Cdc42 and Rac1, which trigger membrane ruffles and lamellipodia and EPEC-induced invasion [143]. EspT exploits the “trigger” mechanism to promote EPEC invasion into nonphagocytic cells via Rac1-dependent membrane ruffle formation [143]. Intriguingly, internalized EPEC mobilizes Tir to the vacuolar membrane, forming intracellular actin rich-pedestals that contribute to intracellular bacterial survival [143]. EspT thus defines an invasive group of EPEC through its ability to manipulate Rho GTPase signalling pathways.

### 5.7. EspL

EspL is well conserved among EHEC strains [151]. EspL is encoded by the *espL2* gene on Sakai prophage-like element 3 (SpLE3) [142], and thereby it is non-LEE encoded effector. EspL is secreted and delivered into host cells in a T3SS-dependent manner, whereas *espL1* seems to be a nonfunctional gene because it does not produce any detectable product or transcript [152]. Furthermore, an *espL* homologue was found in other A/E pathogens, such as EPEC strains, B171 and E2348/69, and *C. rodentium*. It was found that EspL protein is accumulated inside the cells and colocalizes with F-actin near the plasma membrane. Moreover, EspL directly binds F-actin-aggregating annexin 2, increasing its activity. EspL stimulates annexin 2 activity for cross-linking F-actin, while having no apparent direct effect on the F-actin itself [153]. EspL induces F-actin accumulation in the absence of Tir, but this effect is enhanced when combined with the Tir activity. Interestingly, the EspL-induced pseudopod-like protrusion in the host plasma membrane supports colonization by the bacteria, independent of Tir-mediated actin polymerization. EspL supports efficient colonization by increasing annexin 2 ability to aggregate Tir-induced F-actin and by modifying the morphology of the host cell membrane. Thus, EspL directly interacts with annexin 2 at the cytosolic surface of the plasma membrane close to the sites of adherent bacteria and increases annexin 2 F-actin-bundling activity, resulting in the aggregation of F-actin beneath bacterial microcolonies and the formation of pseudopod-like structures [153].

### 5.8. EspV

EspV is the latest T3SS effector reported that modulates actin dynamics [154]. *espV* is not a LEE-encoded effector and is present in ~16% of EPEC and EHEC strains and the expression of EspV in mammalian cells leads to drastic and unique morphological alterations, which are characterized by nuclear condensation, cell rounding, and formation of actin-rich, dendrite-like projections [154]. EspV appears to modulate the actin cytoskeleton by a unique mechanism that is still undefined and expands the activities of cytoskeleton-targeting T3SS effectors beyond Rho GTPase signaling and pedestal formation.

### 5.9. Other Non-LEE Encoded Effectors: *Cif*

Cif (cycle inhibiting factor) was the first identified effector molecule not encoded on the LEE but on a lambdoid phage. Cif is a type III effector molecule translocated into host cells. Cif mediates a G2 cell cycle arrest characterized by inactive phosphorylated Cdk1, resulting in the accumulation of cells with 4N DNA content [155]. Moreover, Cif induces the formation of stress fibers through the recruitment and accumulation of focal adhesions with nuclei and cell enlargement [155]. It has been demonstrated that Cif is sufficient to induce stress fiber formation and G2 arrest [156]. Recently, it has been found that Cif has a catalytic triad strictly conserved and was shown to be crucial for cell cycle arrest, cytoskeleton reorganization and cyclin-dependent kinase inhibitor accumulation [157].

### 5.10. NleA

NleA, a Non-LEE encoded type III translocated bacterial effector protein (also known as EspI), is necessary for the disruption of intestinal TJs by EPEC, in addition to EspF and Map [158]. NleA binds and inhibits COPII, a protein complex involved in trafficking of integral membrane proteins, proteins destined for secretion, or localization to “post-Golgi” compartments such as lysosomes. The effect of NleA on TJ integrity is related to its inhibition of host cell protein trafficking through COPII dependent pathways [159]. This suggests that EspF and Map induce the disassembly of TJs, whereas NleA disruption of COPII function blocks the delivery of new TJ proteins, which would otherwise repair the disassembled tight junctions. Thus, the concerted actions of NleA, EspF, and Map (and perhaps others) are required to cause a stable disruption of tight junctions and the epithelial barrier integrity [158]. NleA has a predicted PDZ-binding domain at its C-terminus, since several PDZ-domain-containing proteins localize into TJs. However, the putative PDZ-binding domain of NleA is not critical in the alteration of epithelial barrier integrity or tight junction disruption [160].

## 6. Diffusely Adherent *E. coli*

Diffusely adherent *Escherichia coli* (DAEC) strains are a heterogeneous group of isolates, all of which exhibit diffuse adherence (DA) to epithelial cells in the classical laboratory assay of adherence to HEp-2 or HeLa cells [161]. The implication of DAEC strains in diarrhea remains controversial [161–163]. Although several cell signaling events have been reported that occur after epithelial cells have been infected by Afa/Dr DAEC (DAEC producing the Afa/Dr adhesin family), the pathophysiological processes that allow intestinal and extraintestinal infections to develop are not fully understood.

**Figure 3:**
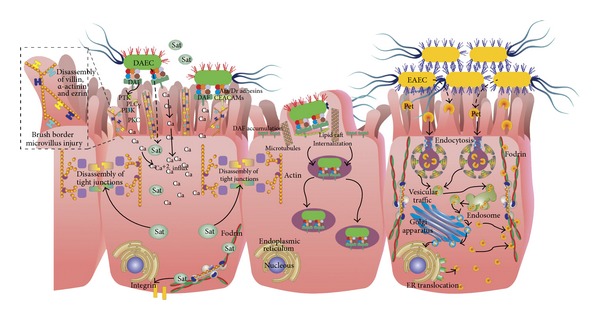
Models of actin cytoskeleton manipulation by enteroaggregative *E. coli* (EAEC) and diffusely adherent *E. coli* (DAEC). EAEC secrete to the serine protease autotransporte of *Enterobacteriaceae* known as Plasmid-encoded toxin (Pet). Pet enters the eukaryotic cell and that trafficking through the vesicular system is required for the induction of cytopathic effects. Thus, after clathrin-mediated endocytosis, Pet undergoes a retrograde trafficking from the Golgi apparatus to the endoplasmic reticulum to be translocated into the cytosol. Where, Pet reaches one of its intracellular target, *α*-fodrin (*α*II spectrin). Pet cleaves epithelial fodrin (between M1198 and V1199 residues), causing fodrin redistribution within the cells; fodrin is a structural link between the plasma membrane and the cortical actin cytoskeleton. On the other hand, Afa/Dr DAEC interact with membrane-bound receptors including the recognition of DAF by Afa/Dr adhesins and of CEACAMs. This receptor recognition develops loss of microvilli, which results from a signaling pathway involving protein tyrosine kinase (PKT), phospholipase C*γ*, phosphatidylinositol 3-kinase (PLC*γ*), protein kinase C (PKC), and an increase in [Ca^2+^]_*i*_. These events control the rearrangements of brush border-associated F-actin and villin cytoskeletal proteins. The elongation of the microvilli also involves the activation of Rho GTPase Cdc42. Secreted autotransporter toxin (Sat) plays an important role in the increased paracellular permeability, which is associated with the reorganization of TJ-associated proteins, ZO-1 and occludin, but does not affect the TER. Internalization occurs in non-polarized epithelial cells via a mechanism involving lipid rafts and dynamic microtubules. Internalized Afa/Dr DAEC bacteria survive within a large, late vacuole (for full details, see main text).

Data accumulated from several studies have suggested that interaction of Afa/Dr DAEC strains with fully differentiated polarized epithelial cells is associated with: (i) brush border lesions resulting from dramatic rearrangements in apical cytoskeleton proteins, (ii) changes in the distribution of tight junction-associated proteins that lead to an increase in paracellular permeability, (iii) secretion of toxin Sat, which induces marked fluid accumulation in the intestine, (iv) induction of mucosal inflammation, and additionally, and (v) the interaction of Afa/Dr DAEC strains with undifferentiated cells show internalization of bacteria by a mechanism involving lipid rafts and microtubules [164, 165] (Figure 3).

### 6.1. Afa/Dr Family of Adhesins and Receptors

The DA pattern of DAEC isolates is due to the production of adhesins encoded by a family of *afa*, *dra*, and *daa* related operons [164], which express the Afa/Dr adhesin complex on the surface of the bacteria. Afa/Dr adhesins (fimbrial and afimbrial) have similar genetic organizations and are expressed by *E. coli* strains of human origin and have been demonstrated to recognize as a receptor the Cromer blood group antigen Dr(a) on the human decay-accelerating factor (DAF, also known as CD55) [164], a characteristic that enables the bacteria to promote agglutination of human erythrocytes even in the presence of mannose.

Bacterial adherence to host cells is the initial step in infections caused by uropathogens or enteropathogens such as *E. coli*. Afa/Dr adhesins mediate bacterial attachment onto target cells by binding to the complement regulatory glycosylphophatidylinositol-(GPI-)anchored protein DAF [166]. DAF, a 70 kDa membrane glycoprotein involved in protecting cells against lysis by homologous complement, is widely distributed in hematopoietic cells, intestinal and urinary epithelia, and endothelial cells [165]. Afa/Dr DAEC strains recognize the short consensus repeat 3 (SCR3) domain of DAF, which plays a pivotal role in the regulatory function of DAF. Afa/Dr adhesins binding results in a dense accumulation of DAF molecules beneath adherent bacteria that could be detected by fluorescent DAF-staining test [167, 168]. DAF is known to have signal transduction capacity; one characteristic feature of GPI-anchored proteins is their lateral membrane mobility, which facilitates coupling to signaling molecules [169]. GPI-anchored molecules associate with tyrosine kinase proteins, which are important regulators of the signal transduction [170]. Furthermore, some carcinoembryonic, antigen-related molecules (CEACAMs) including CEACAM1, CEA, and CEACAM6, also act as receptors for a subfamily of Afa/Dr adhesins (designated as Afa/DrCEA) [171, 172]. This interaction, as well as the binding to DAF [171, 173], induces the recruitment of CEACAM molecules around adhering bacteria [171, 172] in detergent-insoluble microdomains, which is consistent with a role of lipid rafts in the pathogenicity of Afa/Dr strains [174, 175].

Recognition of DAF, CEA, and CEACAM6, but not of CEACAM1, is accompanied by the induction of microvilli extensions at the cell surface, promoting the tight attachment of bacteria. Signaling controls the induced microvilli extensions.

### 6.2. Brush Border Microvillus Injury

DAEC strains promote structural and functional injuries in the intestinal cells, including the recognition of DAF in infected epithelial cells by Dr and F1846 adhesins and which is followed by microvillus injury. Brush border lesions result from dramatic rearrangements in apical cytoskeleton proteins, such as F-actin, villin, *α*-actinin, ezrin and occasionally tropomyosin, proteins that play a pivotal role in the organization and maintenance of brush border integrity [163, 173, 176]. At the same time, it has also been observed an alteration in the distribution of functional brush border-associated proteins controlling the absorption/secretion function. In cultured intestinal cells forming a monolayer mimicking an epithelial intestinal barrier, Afa/Dr DAEC infection is followed by brush border lesions characterized by an injury of microvilli, evidenced by dramatic changes in the architecture of the microvilli limited to the point of bacterial contact, showing disruption of the tip of the microvilli and then nucleation [163].

The brush border lesion is the consequence of the disassembly of two major cytoskeleton proteins, F-actin, and villin as a result of the activation of Ca^2+^-dependent signaling. This phenomenon involves the activation of a cascade of signaling coupled to the glycosylphosphatidylinositol-anchored receptor of the adhesin, without bacterial cell entry [176, 177]. This signal pathway involves protein tyrosine kinase, phospholipase C*γ*, phosphatidylinositol 3-kinase, protein kinase C, and Ca^2+^ [177]. In turn, the loss of brush border results from defective expression of brush border-associated functional intestinal proteins, including sucrose-isomaltase (SI), dipeptidylpeptidase IV (DPPIV), glucose transporter SGLT1, and fructose transporter GLUT5 [176].

### 6.3. Lesions in Tight Junctions

Afa/Dr DAEC infection also leads to an increase in paracellular permeability and dramatic rearrangement of the distribution of the tight junction (TJ)-associated proteins, ZO-1 (which, as mentioned before, is linked to the cytoskeleton and plays a pivotal role in the TJ architecture) and occludins (which are important in the sealing of the TJ), without affecting the transepithelial electrical resistance of the cell monolayer [178]. However, the distribution of the zonula adherens-associated E-cadherin is not affected. Functional alterations in TJs are independent of the C1845-induced apical cytoskeleton rearrangements, indicating that pathogenic factor(s) other than F1845 adhesin may be operanting in Afa/Dr DAEC C1845 infection [179].

Additionally, there is evidence that the secreted autotransporter toxin (Sat) belonging to the subfamily of serine protease autotransporters of *Enterobacteriaceae* (SPATEs) acts as a virulence factor in Afa/Dr DAEC. This matter will be discussed in the next section.

### 6.4. Internalization of Afa/Dr DAEC

Several reports have indicated that various Afa/Dr-positive strains are able to enter epithelial cells *in vitro* [174, 180–182]. Afa/Dr DAEC enter epithelial cells by a zipper-like mechanism [175, 180], but to a lesser extent than that achieved by invasive bacteria such as *Salmonella*; thus, they are not true invasive pathogens, since only a small percentage of bacteria adhering to the cells are internalized. To enter undifferentiated epithelial cells, adhering Afa/Dr DAEC cells promote plasma membrane projections to form a zipper-like structure that engulfs the adhering bacteria [175]. These events resemble that of the uptake of *Neisseria*, where exist distinct mechanisms of internalization of *Neisseria gonorrhoeae* by members of the GPI-anchored CEACAM receptor family involving Rac1- and Cdc42-dependent and -independent pathways [183].

The bacterial factors involved in Afa/Dr DAEC internalization have not been clearly identified, but DraE or AfaE adhesin subunits are necessary and sufficient to promote the receptor-mediated bacterial internalization [184]. Internalization of Dr-positive bacteria occurs by a zipper-like mechanism which is independent of the Dr-induced mobilization of the microfilament cytoskeleton and of the signaling molecules that control the Dr-induced F-actin rearrangements. The lipid raft-dependent internalization of Dr-positive bacteria occurs in both cells expressing or no expressing the marker VIP21/caveolin of caveolae, but cholesterol is critical for bacterial internalizatio [175, 184].

## 7. Actin Cytoskeleton Disruption by Serine Protease Autotransporters

Sat from DAEC, Pet from EAEC, and EspC from EPEC belong to the SPATE subfamily of autotransporters [185]. SPATEs and other autotransporters use a type V secretion system for export to the extracellular space [185, 186]. The autotransporters contain all of the information necessary for passage through the inner membrane as well as the outer membrane. To mediate its own secretion, an autotransporter contains three functional domains: an N-terminal signal sequence, an extracellular passenger domain, and a C-terminal *β*-barrel domain. The signal sequence initiates Sec-dependent transport across the inner membrane and is proteolytically removed in the periplasmic space. The C-terminal domain forms a *β*-barrel pore in the outer membrane, which facilitates delivery of the passenger domain to the extracellular space. The passenger domains of some autotransporters remain anchored to the extracellular face of the outer membrane, but SPATEs are released from the bacterial cell by proteolytic nicking of a site between the *β*-barrel pore and the passenger domain. The mature, secreted SPATEs are 104–110 kDa toxins that contain a typical N-terminal serine protease catalytic domain followed by a highly-conserved *β*-helix motif, which is present in nearly all autotransporters [185–187]. Although the general process of SPATE secretion is understood, the details of many events in SPATE biogenesis (chaperone function in the periplasm, mechanism of *β*-barrel insertion into the outer membrane, translocation pathway across the outer membrane, proteolytic release of the mature protein from the outer membrane, etc.) remain unresolved [185].

It has been proposed that SPATEs can be divided phylogenetically into two distinct classes, designated 1 and 2 [188]. Class 1 SPATEs are cytotoxic *in vitro* and induce mucosal damage on intestinal explants. Although the actions of class 1 SPATEs are not fully understood, several have been shown to enter eukaryotic cells and to cleave cytoskeletal proteins [189–191]. While the class 2 SPATEs induces mucus release, cleaves mucin, and confers a subtle competitive advantage in mucosal colonization [192–194].

The class 1 SPATEs secreted by *E. coli* pathotypes include Sat, Pet, and EspC (Figures 3 and 2). Interestingly, despite their high level of homology, the SPATE proteins demonstrate distinct mechanisms of internalization and interaction with the cytoskeletal proteins. For instance, EAEC and EPEC use distinct mechanisms of toxin delivery (Pet and EspC, resp.) to the host cell. Pet is internalized by receptor-mediated endocytosis, followed by a retrograde transport to reach the cytosol [195, 196], whereas EspC is internalized by a mechanism that requires EPEC contact with the host cell and the production of the type III secretion system [197, 198]. After gaining access to the epithelial cytosol, both Pet and EspC target the actin-binding protein fodrin (also known as spectrin) [190, 199]. However, Pet and EspC modify fodrin by different mechanisms. Fodrin is an elongated heterodimer consisting of a 280 kDa *α* subunit and a 246 kDa *β* subunit [200, 201]. Two heterodimers associate in a head-to-head orientation to form a functional tetramer. Both fodrin subunits contain multiple copies of a 106 amino acid motif termed the spectrin repeat as well as a src homology 3 domain, a pleckstrin homology domain, a calmodulin binding domain, and an actin-binding domain. Through these domains, the fodrin tetramer anchors membrane lipids and transmembrane proteins to the cortical actin cytoskeleton, which lies beneath the plasma membrane. The interaction between fodrin and filamentous actin provides a degree of structural organization to the actin cytoskeleton, which helps the cell withstand mechanical stress. Fodrin is also involved in epithelial morphogenesis [202] and apoptotic cell death, when it is cleaved in the 11th repetitive unit by calpain or caspase-3, [203, 204].

Pet displays affinity for *α*-fodrin *in vitro* and cleaves epithelial fodrin *in vivo* [190, 205]. Two breakdown products of 37 kDa and 72 kDa were generated from *in vitro* Pet activity against a recombinant GST-tagged construct that contained a 109 kDa fragment of *α*-fodrin representing the 8th to the 14th spectrin repeats (codons 809–1529) [190]. This was the first report showing the cleavage of *α*-fodrin by a bacterial protease. The cleavage occurs between residues M1198 and V1199 within the calmodulin-binding domain of fodrin 11th repetitive unit. Site-directed mutagenesis of these amino acids prevented GST-fodrin degradation by Pet. An inactivating S260I mutation in the Pet serine protease motif also prevented the proteolysis of GST-fodrin. Pet also cleaves epithelial fodrin in cultured cells [190, 205]. *In vivo* proteolysis of fodrin did not occur in the presence of PMSF (a serine protease inhibitor) or with the Pet S260I mutant toxin. Pet activity against fodrin generated a 120 kDa breakdown product, which was found in intracellular aggregates as membrane blebs. Loss of fodrin disrupts the structural link between the plasma membrane and the cortical actin cytoskeleton. Contraction of the actin cytoskeleton, loss of actin stress fibers, cell rounding, and eventual detachment from the substratum resulted from the loss of proper actin architecture. In intestinal epithelial cells, fodrin proteolysis may also lead to disassembly of the microvilli; the lower part of the actin filament bundle in the microvilli core is anchored and stabilized by a specialized structure that contains a dense core of fodrin [206]. Cleavage of *α*-fodrin to a 120 kDa fragment has been detected in apoptotic cells [203, 204], then Pet activity may contribute to enterocyte death by triggering an apoptotic pathway.

Like Pet, EspC cleaves GST-fodrin in a reaction that requires a functional serine protease motif [199]. However, unlike Pet, fodrin proteolysis by EspC generates four subproducts with apparent molecular masses of 72, 43, 45, and 34 kDa. The last two fragments come from further processing of an initial fodrin proteolytic fragment of around 72 kDa, suggesting the existence of two cleavage sites: fodrin's 11th and 9th repetitive units. The recognition of separate fodrin proteolytic sites by Pet and EspC was further emphasized by competition studies using the inactive Pet S260I and EspC S256I mutant toxins [199]. These nonfunctional toxins are able to enter epithelial cells but are unable to cleave fodrin or damage the actin cytoskeleton. An excess of EspC S256I blocked the cytoskeletal damage caused by wild-type EspC, but it did not block the cytoskeletal damage caused by Pet. Moreover, an excess of Pet S260I did not prevent the cytoskeletal damage caused by wild-type EspC. The lack of competition between Pet and EspC consequently indicated that the two toxins bind to separate regions of fodrin. These data collectively demonstrated that Pet and EspC recognize different binding and cleavage sites in fodrin. The distinct patterns of fodrin proteolysis by Pet and EspC result in distinct cellular effects. Both toxins generate cytopathic and enterotoxic effects through disruption of the actin cytoskeleton. However, fodrin degradation by EspC is not accompanied by redistribution of the proteolytic fragments to membrane blebs [199]. Furthermore, the fodrin proteolytic fragments generated by EspC do not correspond to the 120 kDa breakdown product resulting from Pet activity against the calmodulin-binding domain of fodrin [190, 205]. EspC cleavage sites occur outside of the calmodulin-binding domain, although one cleavage site is close to this domain. Since calmodulin and calpain I coordinately regulate the interaction between fodrin and filamentous actin to maintain the cytoskeletal integrity [207], the fact that Pet but not EspC cleaves within the calmodulin-binding domain of fodrin is perhaps related to the different cellular effects elicited by the two toxins. The cleavage of fodrin by calcium-dependent proteases has also been observed in several cellular processes, and this proteolysis can lead to various necrotic and apoptotic events [208]. In a similar manner, the differential cleavage of fodrin by Pet and EspC could trigger different biological events in addition to the shared disruption of filamentous actin.

Sat (which is also secreted by uropathogenic *E*. *coli*) enters host cells by an unknown mechanism and localizes to the cytoskeletal fraction, where it can cleave target proteins such as spectrin (fodrin) and integrin. The cytoskeletal effects mediated by Sat on urinary epithelial cells are likely associated with the degradation of fodrin (nonerythrocyte spectrin). Fodrin/spectrin is involved in stabilizing membrane structures, maintaining cell shape, and linking actin filaments with the plasma membrane [201, 202, 206]. The results of Maroncle et al. [209] affirmed that Sat is able to degrade both *α* and *β*-spectrin chains as previously demonstrated by Dutta et al. [188] and similarly shown for Pet and EspC [199, 210]. Proteolytic attack on fodrin, thereby altering the cytoskeleton, may explain the rounding, elongation, membrane ruffling, and detachment observed when urinary cells are treated with wild-type and revertant Sat. Sat also acts as virulence factor in Afa/Dr DAEC by promoting lesions in the tight junctions of polarized epithelial Caco-2/TC7 cells by inducing rearrangements of the TJs-associated proteins ZO-1, ZO-3, occluding, and claudin-1 [211]. Guignot et al. [211] demonstrated that despite the fact that Afa/Dr DAEC expresses Sat, this toxin is not involved in the Afa/Dr DAEC-induced alteration of the brush border-associated F-actin cytoskeleton in intestinal cells [211].

## 8. Conclusion

Pathogenic microbes, such as pathogenic *E. coli*, subvert normal-cell processes to create a specialized niche, which enhances their survival. A common and recurring target is the cytoskeleton, mainly the actin cytoskeleton, since the F-actin filaments are highly dynamic structures, whose supramolecular organization is constantly modified according to cellular needs. Actin dynamic behavior is regulated by a large number of binding proteins, which drive intracellular and extracellular signaling pathways. It is therefore not surprising that the actin cytoskeleton is one of the main targets of bacterial proteins, and a key role for the host-pathogen interaction. Microbes utilize the host cell cytoskeleton for cell attachment, entry into cells, movement within and between cells, vacuole formation and remodeling, and avoidance of phagocytosis. In order to accomplish these processes, bacteria secrete and inject toxins and effectors to hijack the cell machinery. It is clear that the biology of the different *E. coli* pathotypes is complex, since each pathotype has a distinct subset of genes involved in the subversion of host responses and hijacking of host cell machinery. Furthermore, in many pathotypes, the same machinery or process is target but the mechanism and outcome are different. It will be easy to imagine that the effects of some effectors on the actin dynamics cause structural changes on the cell morphology that allow to other effector proteins to better reach their molecular target. For instance, for pedestal formation Tir play a relevant role as an adaptor for actin polymerization, but for pedestal maturation and growing EspF has to remove tight junction proteins, which are recruited into the pedestal. These two later events have to take place in order to expose integrin proteins at the basolateral side of the epithelial cells, which can be used as receptors, as well as to cause luminal channel mislocalization. All these events results the final outcoming during a specific pathotype infection. A specific pathotype can produce many bacterial effectors, which can create an intricate interaction circuit that synergize, regulate, inhibit, or have redundant functions, and all together occur for the proprietary benefit of the pathogen. Most of these effectors have a modular structure including different motifs that mimic the function of these large actin-binding proteins and regulatory factors of actin dynamics such as Rho GTPases, Nck, N-WASP, among others.

Bacterial pathogenesis is a quick evolving and expanding field. Genetics, genomics, and proteomics efforts continue to identify more potential virulence factors, but our understanding of the interactions between virulence factors (i.e., effectors and toxins) and host components remain incomplete. It is a considerable challenge to integrate the numerous targets and effectors, and to integrate this knowledge into an accurate understanding of the mechanisms by which effector proteins cause diseases. Furthermore, our increased understanding of these processes in recent years, as well as in the next years will contribute to greater our comprehension of the molecular causes of infectious diseases, and also to increase our knowledge of cell biology.
